# Supplementary material for: Structure prediction of alternative protein conformations
Source: Nat Commun. 2024 Aug 26;15:7328. doi: 10.1038/s41467-024-51507-2 (PMC11347660; doi:10.1038/s41467-024-51507-2)
Supplement: Supplementary file 1 — Supplementary Information [file 41467_2024_51507_MOESM1_ESM.pdf]

Supplementary information

Supplementary Table 1. The biological relevance of sampled conformations (what induces the conformational switch).

Train id	Test id	Reason for switch	Category (ligand binding, protein binding, introduced mutations, unclear)
1DPE	1DPP	transport, chemotaxis	ligand binding
1E8C	7B68	substrate binding	ligand binding
1IGV	4ICB	allostery from receptor binding	protein binding
1IWL	7Z6W	lipoprotein binding	protein binding
1L5T	1BKA	ligand (anion) binding	ligand binding
2BBW	2AR7	substrate binding	ligand binding
2C78	1HA3	binding of different ligands	ligand binding
2DRI	1URP	substrate binding	ligand binding
2H2Z	2QCY	mutated sites	introduced mutations
2HF2	1RLM	unclear	unclear
2QW1	1GLG	mutated sites	introduced mutations
2WRZ	8ABP	substrate binding	ligand binding
2ZGZ	1MWK	substrate binding	ligand binding
3C6Q	2H3H	substrate binding	ligand binding
3DKC	8ANS	binding of different ligands	ligand binding
3IIA	3PLQ	binding of inhibitor	ligand binding
3IUN	3IVM	substrate binding	ligand binding
3K6U	3K6X	substrate binding	ligand binding
3L6H	3L6G	substrate binding	ligand binding
3O9P	4TOZ	substrate binding	ligand binding
3OO6	3OO9	substrate binding	ligand binding
3ROI	3SLH	substrate binding	ligand binding
3ZSF	2YLN	substrate binding	ligand binding
4A3P	3T9L	mutated sites	introduced mutations
4AVB	4AVA	binding of different ligands	ligand binding
4KQP	6H30	binding of different ligands	ligand binding

4P0I	5OVZ	substrate binding	ligand binding
4PUT	4KA8	enzyme activity	unclear
4QIQ	4GC0	substrate binding	ligand binding
4ZYR	2V8N	substrate binding	ligand binding
5AYN	5AYO	substrate binding	ligand binding
5LE6	5LE3	mutated sites	introduced mutations
5TUJ	5T0W	substrate binding	ligand binding
6CVA	6DZX	substrate binding	ligand binding
6E9O	6E9N	substrate binding	ligand binding
6FHZ	4MLB	substrate binding	ligand binding
6HKR	7OXW	substrate binding	ligand binding
6HNI	6HNK	substrate binding	ligand binding
6JAQ	6JAL	substrate binding	ligand binding
6O1X	6O1Z	substrate binding	ligand binding
6P8O	6P8R	protein binding	protein binding
6S2U	7OHG	substrate binding	ligand binding
6S9O	4PLQ	mutated sites	introduced mutations
6TG3	5L9P	substrate binding	ligand binding
6UHS	6UHI	substrate binding	ligand binding
6XLY	3ZUK	binding of inhibitor	ligand binding
6YED	6YE8	substrate binding	ligand binding
7CML	3E7O	substrate binding	ligand binding
7DE3	2C95	substrate binding	ligand binding
7SY9	8DP2	substrate binding	ligand binding
7VER	7VEV	substrate binding	ligand binding
8DP7	8DP6	substrate binding	ligand binding

Rescuing failed predictions with increased sampling for the MSA clustering procedure

To see if it is possible to “rescue” failed predictions by increasing the number of recycles and samples, we select 10 examples that are predicted with TM-score<0.8 at random. We increase the number of recycles to 10/20 and take 50/100 samples per cluster size with the MSA cluster procedure (Methods). **Supplementary Figure 1**a shows the previous best TM-scores towards the test conformations vs. the best with the increased sampling and recycling. Increasing the number of recycles or samples has a negligible effect on the

outcome, with only one of the targets displaying an improvement to a TM-score>0.8 and all scores are only moderately improved.

Supplementary Figure 1b shows the best TM-score and the number of clusters used to obtain this in the rescue attempt. There is no apparent relationship between TM-score and cluster size and the only successful example is obtained at a small cluster size of only 32 sequences. Therefore, exploring the more expensive sampling settings with thousands of sequences, more recycles or samples appears unproductive. **Supplementary Figure 1**c shows the distribution of cluster sizes using the best TM-scores towards the test set for the successful predictions (n=81, Table 1). Increasing the number of clusters does not appear to be beneficial in most cases as most high scores are found at cluster sizes <100.

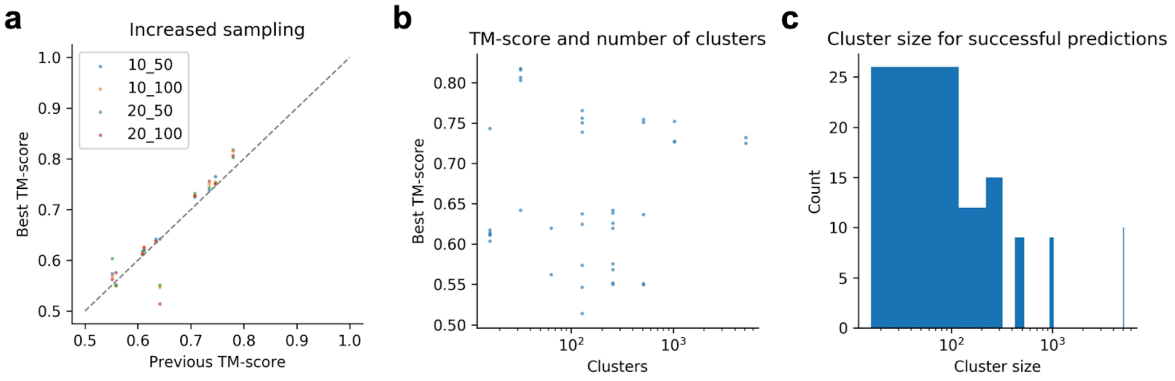

Supplementary Figure 1. a) The best TM-scores obtained previously using 3 recycles and 13 samples per cluster size vs. 10/20 recycles and 60/100 samples per cluster size. Ten structures from the test set that were unsuccessful (TM-score<0.8) were used here with PDB IDS: 2NRV, 4NTJ, 5WU4, 4WXX, 6WBO, 5LJ8, 1M61, 4WTV, 2E1R and 6YHK. Increasing the recycles or the number of samples has a negligible effect. **b)** TM-score and number of clusters for the rescue set. Using more clusters does not seem to be beneficial. **c)** MSA cluster size distribution for the best TM-scores towards the test set using the successful predictions (n=81, Table 1). Using more clusters does not appear to be beneficial in most cases as most high scores are found at cluster sizes <100.

Rescuing failed predictions with increased sampling for the dropout procedure

MSA clustering proved more successful than dropout. To analyse if proteins that are successful with clustering but not with dropout **(n=8 structures)** are a result of too few samples being taken we increased the number of samples to 1000. We find that the increased sampling does not improve the results as none of the predicted structures obtain TM-scores above 0.8 to both train and test conformations.

Supplementary Table 2. Best TM-scores from 1000 samples taken with the dropout strategy for eight cases where the MSA clustering strategy succeeds but the dropout does not.

Train id	Test id	TM-score train	TM-score test	Successful
2BBW	2AR7	0,7897	0,88173	FALSE
2H2Z	2QCY	0,78848	0,81724	FALSE
4A3P	3T9L	0,89536	0,78883	FALSE
6XLY	3ZUK	0,76138	0,9587	FALSE
6TG3	5L9P	0,93933	0,74851	FALSE
5LE6	5LE3	0,66259	0,8428	FALSE
6O1X	6O1Z	0,76745	0,87056	FALSE
6S2U	7OHG	0,86048	0,77595	FALSE

Favourable conformations

In some cases, seen (train) conformations are sampled more and in others the unseen (test) are more favoured. To analyse why this is, we selected five examples where the train TM-scores are higher and five where the test TM-scores are (Supplementary Figures 2a and b). We analysed the conformational types and the biological relevance of the selected conformations.

All conformations belong to the ‘rearrangement’ category (Figure 2) suggesting that the type of conformational change does not determine the outcome. The average number of amino acids for the proteins when the train conformations are better is 344 vs 318 when the test is, suggesting that protein size plays a role. We also analysed the biological relevance of these changes and concluded that 4 are ligand binding and one belongs to the category of introduced mutations when the train conformations are better. When the test conformations are better, three are ligand binding, one is unclear and one is due to introduced mutations.

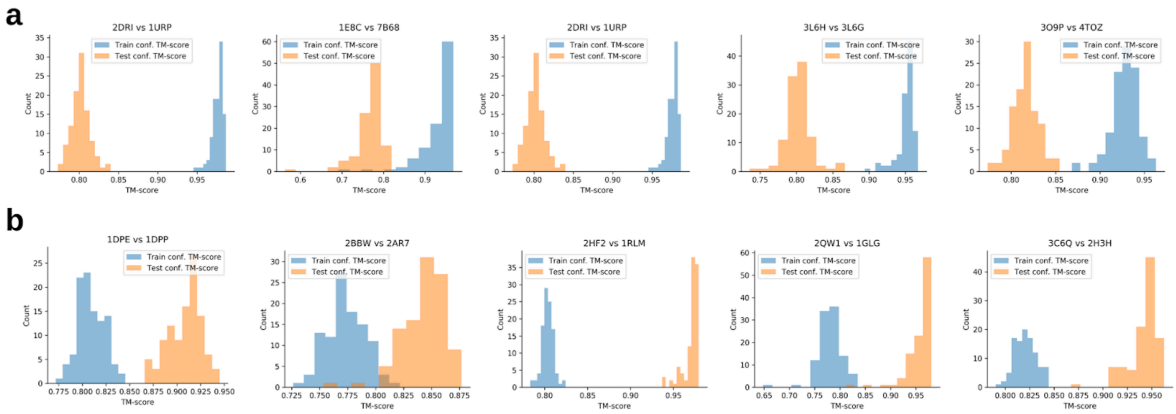

Supplementary Figure 2. Distributions (n=104 per distribution) of cases where either the train **(a)** or test **(b)** conformations are more favourable.

Comparison with NMR ensembles

Cfold was not trained on any NMR structural data. To see if structural fluctuations observed in NMR ensembles can be sampled, we selected three NMR ensembles with structural fluctuations from the PDB to investigate the possibility of sampling similar fluctuations with Cfold. We ran Cfold with the clustering strategy (Methods) for PDB IDs 2M6Q, 2N4A and 7ZK0. Supplementary Figure 3 displays the resulting ensembles. We also analyse the variation through principal component analysis (PCA) [35] and display the samples on the first two PCs. We perform PCA on all coordinates in each structure after structurally aligning them.

For 2M6Q, Cfold does not capture the structural variation of the loop regions. 2N4A shows little variation and here the Cfold PCA space is similar to that of 2M6Q. Most Cfold predictions are highly similar as can be seen by the high concentration of points around (0,0) in the PCA projection. For 7ZK0, the Cfold predictions vary substantially while the NMR ensemble only shows variation in loop regions. In conclusion, Cfold samples tend to either over- or underestimate the variation observed in NMR ensembles. We note that Cfold is not intended for sampling protein dynamics, but to predict distinct conformational states.

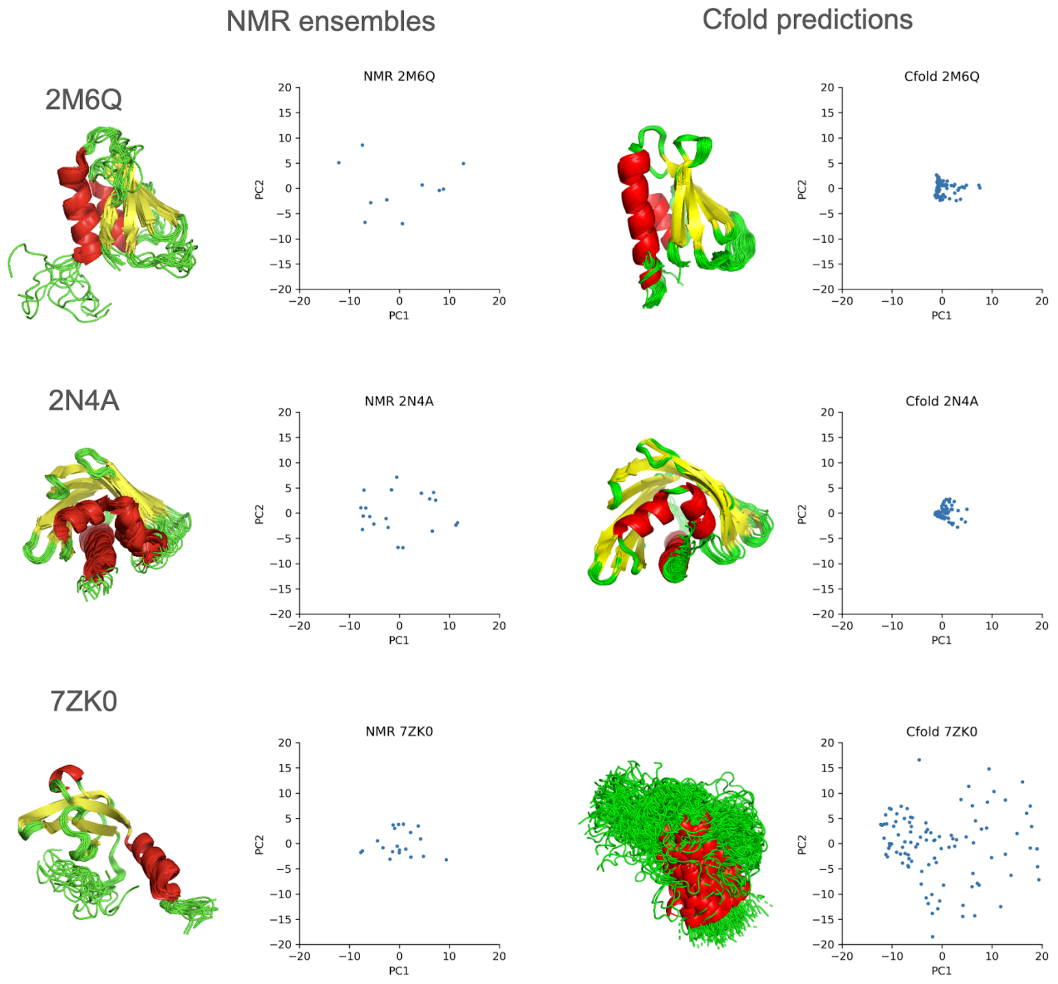

Supplementary Figure 3. Comparison of NMR ensembles and Cfold samples for three structures with PDB IDs 2M6Q, 2N4A and 7ZK0 (10, 20 and 20 NMR structures respectively). The structural variation is visualised with PCA on the first two PCs.

Supplementary Table 3. Number of sequences and structural clusters in the training, validation and test partitions. The procedure for selection and generation of these is outlined in “Proteins with alternative conformations in the PDB”.

Partition	Number of sequences	Fraction of sequences	Number of structural clusters
Training	56407	87.85%	6157
Validation	3539	5.51%	317
Test	4263	6.64%	222
Total	64209	100%	6696

Supplementary Table 4. Network architecture. Number of blocks and cluster sizes used in the network.

**Component**	**Size**
Evoformer blocks	48
MSA clusters	128
Extra MSA clusters	1024
Structure module blocks	8
